# Treatment of posttraumatic stress disorder (PTSD)

**DOI:** 10.1080/19585969.2026.2680838

**Published:** 2026-06-12

**Authors:** Borwin Bandelow

**Affiliations:** Department of Psychiatry and Psychotherapy, University Medical Center, Göttingen, Germany

**Keywords:** Posttraumatic stress disorder, treatment, medication, psychotherapy

## Abstract

The present review summarises recommendations for the management of posttraumatic stress disorder (PTSD) based on a consensus among international experts in the field.

Cognitive-behavioural therapy (CBT) has the best evidence base as a psychotherapy modality. Virtual reality exposure therapy appears to be an emerging treatment option for PTSD. Eye Movement Desensitisation and Reprocessing (EMDR) therapy and digital CBT-based interventions did not demonstrate superiority compared to controls. Controlled studies supporting the use of psychodynamic therapy are lacking. Early prophylactic psychotherapy for trauma victims is ineffective and may be harmful.

First-line pharmacotherapy includes selective serotonin reuptake inhibitors (SSRIs) and the serotonin-noradrenaline reuptake inhibitor (SNRI) venlafaxine. Several second- and third-line medications are available for treatment-refractory patients. Currently, no medications can be recommended for prophylactic use in trauma victims.

Although evidence is incomplete regarding combined psychotherapy and pharmacotherapy, available studies generally favour combination treatments over monotherapy. Repetitive transcranial magnetic stimulation (rTMS) showed efficacy in one study. In children and adolescents with PTSD, CBT demonstrated a medium effect size compared to active controls, while studies on SSRI treatment in this population yielded inconsistent results.

## Introduction

Posttraumatic stress disorder (PTSD) is a severe mental health condition that can develop as a consequence of exposure to traumatic events. The world-wide lifetime prevalence is estimated to be 3.9% in the general population and 5,6% among those with trauma exposure (Koenen et al. 2017).

PTSD may manifest acutely following a traumatic event but can also have a delayed onset occurring months or years afterward. Delayed onset, defined as onset more than six months after the trauma, has been reported in approximately 20% to 25% of cases (Bryant et al. 2013) but rarely develops without any symptoms bridging the interval between the trauma and full PTSD onset (Bonde et al. 2022).

Symptoms of PTSD tend to improve over time unless repeated triggering factors occur. Of those who develop PTSD, one-third recover within the first six months, and another third within two years. Only one-third, or 3–4% of those who have been exposed to a potentially traumatic event, develop chronic PTSD (Fabra et al. 2009).

PTSD is commonly comorbid with depression, substance use and anxiety disorders (Kessler et al. 2005).

## Aetiology of PTSD

Experiencing severe traumatic events does not invariably lead to the development of posttraumatic stress disorder (PTSD). In a large sample of American adults, it was demonstrated that although 61% of men and 51% of women were exposed to a traumatic event at some point in their lives, the lifetime prevalence of PTSD was 8% in men and 20% in women (Kessler et al. 1995). PTSD prevalence varies significantly by type of trauma experienced. Conditional risks were found to be 30% to 53% for sexual assault/rape (Kessler et al. 1995; Breslau et al. 1998), 46% for kidnapping (Favaro et al. 2000), 34% for being injured in a terrorist attack (North et al. 1999), 22% for exposure to combat (Tedla et al. 2024), 10% to 15% for accidents (Kessler et al. 2017), 12% for unexpected death of a loved one (Kessler et al. 2017) and 5% to 10% for natural disasters (Norris et al. 2002).

Although the aetiology of PTSD is associated with traumatic experiences, individuals with specific predispositions may exhibit more pronounced responses to trauma. Pretraumatic factors that have been associated with increased rates of PTSD include a history of mental disorders (e.g. alcohol or substance abuse, depression, anxiety disorders, previous PTSD), family psychiatric history, childhood adversity (family dysfunction, parental separation or death), female gender, being a member of a sexual minority, personality factors (e. g. neuroticism, hostility) and social ecological factors. Adverse factors that may promote persistent PTSD include subsequent adverse life events, financial or other trauma-related losses, repeated upsetting reminders and lack of social support (Biederman and Faraone 2005; DiGangi et al. 2013; Sayed et al. 2015).

The disposition to develop PTSD may be influenced by genetic and neurobiological factors. Twin studies have estimated the heritability for PTSD symptoms to be 30% to 70% (True et al. 1993; Stein et al. 2002; Sartor et al. 2012). A large number of studies investigated the neurobiology of PTSD (Bandelow et al. 2017; Al Jowf et al. 2023). Research fields include: brain imaging, brain circuit disruption, neurotransmitter dysregulation (e. g. serotonergic dysregulations), hypothalamic–pituitary–adrenal (HPA) axis dysfunction, and brain physiology (e.g. EEG alterations). However, despite extensive research efforts, the search for reliable biological markers of PTSD has thus far been unsuccessful.

## Diagnosis

The definition of PTSD is given in Table 1. Diagnosis of PTSD should be based on the criteria of DSM-5 TR (APA 2022) or ICD-11 (ICD-11 2022).

**Table 1.** Symptoms of PTSD according to the DSM-5 (APA 2013) (abbreviated).

1. Intrusion: Recurrent, involuntary, and distressing memories of the traumatic event; nightmares related to the trauma; dissociative reactions (“flashbacks”) where the individual feels as if the traumatic event is recurring; distress at exposure to cues that resemble the traumatic event; physiological reactions to reminders of the trauma.
2. Avoidance Symptoms: avoidance of memories, thoughts, or feelings about the traumatic event; avoidance of external reminders (e.g., people, situations) that arouse distressing memories.
3. Negative Alterations in Cognitions and Mood: inability to remember an important aspect of the traumatic event (dissociative amnesia); negative beliefs or expectations about oneself, others, or the world; distorted cognitions about the cause or consequences of the traumatic event that lead the individual to blame themselves or others; negative emotional state (e.g. fear, anger, guilt, shame); diminished interest in significant activities; feelings of estrangement from others; inability to experience positive emotions (e. g., happiness, satisfaction, or loving feelings).
4. Alterations in Arousal and Reactivity: irritability and angry outbursts (with little or no provocation), verbal or physical aggression; reckless or self-destructive behaviour; hypervigilance; exaggerated startle response; problems with concentration; sleep disturbances.
These symptoms must last for more than 1 month and cause significant distress or impairment in social, occupational, or other areas of functioning. The disturbance should not be attributable to a substance or another medical condition.

Distinguishing PTSD as a pathological response from a normative or adaptive reaction to severe trauma can be challenging. Because diagnosis relies exclusively on patients’ self-report, it may be difficult to differentiate full-syndrome PTSD from subclinical presentations, such as normative grief reactions. Overdiagnosis in individuals exposed only to minor traumatic events risks trivialising the disorder, while overly stringent diagnostic thresholds may result in withholding treatment from those truly afflicted.

PTSD must be differentiated from other trauma-related disorders, such as Acute Stress Disorder and Adjustment Disorder. In addition, it is essential to distinguish PTSD from other psychiatric conditions, including major depression and anxiety disorders.

ICD-11 introduced a new diagnostic entity termed “Complex PTSD”. This disorder meets all criteria for PTSD and is further characterised by (1) difficulties in affect regulation, (2) negative self-concept with feelings of worthlessness, shame, or guilt related to the traumatic event, and (3) problems in sustaining relationships. Because all these symptoms can also be present in “simple” PTSD, differential diagnosis may be challenging, and it has been suggested that the existing evidence does not support the establishment of this new category (Resick et al. 2012). There has also been debate as to whether “Complex PTSD” is actually PTSD with comorbid borderline personality disorder (BPD) (Karatzias et al. 2023). However, PTSD can often be easily distinguished from BPD, as the symptomatology is different and the diagnosis of PTSD requires a severe trauma, whereas the BPD can occur without traumatic life events.

In the diagnostic assessment of PTSD, the potential presence of malingering, defined as the deliberate exaggeration or fabrication of PTSD symptoms, should be considered. Common motives include seeking financial compensation, obtaining sick leave or disability status, or garnering sympathy and support. Prevalence rates of malingering vary by context, with approximately 7.4% reported in nonforensic evaluations (Rogers et al. 1994) and rates ranging from 16% to 53% in forensic settings (Rogers et al. 1994; Stevens et al. 2013). Malingering should be considered when a patient seeks financial compensation, there is a discrepancy between the severity of the traumatic event and the reported symptom severity, previous treatments have been reported as ineffective or even harmful, or when no treatment has been sought. Additionally, in clinical trials involving individuals with PTSD, it is crucial to exclude malingerers, as this subgroup tends to report no improvement regardless of intervention, which may result in an underestimation of the efficacy of the treatment under investigation.

## Treatment

PTSD can effectively be treated with psychotherapy and medications. In this paper, the available evidence from clinical studies for PTSD is summarised.

It is important to recognise that not all individuals exposed to trauma require treatment. Many demonstrate remarkable resilience and naturally regain psychological stability following distressing events.

### Methods

The recommendations in this review are derived from the Guidelines for Treatment of Anxiety, Obsessive-Compulsive and Posttraumatic Stress Disorders (Version 3) of the World Federation of Societies of Biological Psychiatry (WFSBP) (Bandelow et al. 2023b). For this initiative, a consensus group of 33 international experts reviewed the existing evidence from randomised controlled trials (RCTs) for the treatment of PTSD (*n* = 234) in adults and children. The review process is described in Bandelow et al. (2023a).

The recommendations for drug treatment are based on RCTs comparing the drug with placebo or an established reference drug.

Psychotherapy trials often use waitlist conditions as a control, while medications are tested against placebo. A waitlist control group can only demonstrate that a specific treatment is more effective than no intervention at all, but it cannot establish that this particular treatment is more effective than a simple conversation without the application of a specialised psychotherapeutic technique. Moreover, in a waitlist trial, patients are not blind to their treatment allocation. Last, waitlist is an easy target to beat. Therefore, in psychotherapy research, an “attention placebo” oder “psychological placebo” is used as a control group, in which individuals without formal psychotherapy training conduct sessions of the same number and duration as those in genuine psychotherapy, engaging patients in discussions about their problems. This would be the equivalent of the pill placebo used in pharmacological studies.

To keep the conditions for psychotherapy and medications comparable, the evidence for the efficacy of psychotherapeutic interventions in our analysis was based only on studies comparing psychotherapy with a psychological placebo or another form of active control group (i. e., pill placebo, treatment as usual, or psychoeducation). Studies using a waitlist were also analysed; however, they were not taking into account when determining the efficacy of a certain psychological treatment.

When there was ambiguous evidence for a certain treatment, e.g. when the number of positive and negative studies was almost equal, the decision was based on a meta-analysis of these studies. The method of these meta-analysis was adopted from Bandelow et al. (2015).

Table 2 presents the World Federation of Societies of Biological Psychiatry (WFSBP) grading system (Hasan et al. 2019). First, the level of evidence (LoE) was determined for each treatment. Second, a recommendation grade (RG) was assigned, which considered not only treatment efficacy but also the risk-benefit ratio.

**Table 2.** WFSBP grading system: levels of evidence (LoE) and recommendation grades (RG) (Hasan et al. 2019).

Levels of evidence (LoE)			
Positive evidence		Negative evidence	
A	Strong evidence for the intervention	A-	Strong evidence against the intervention
B	Limited evidence for the intervention	B-	Limited evidence against the intervention
C	Weak evidence for the intervention	C-	Weak evidence against the intervention
D	No evidence		
Recommendation grades (RG)			
**Positive recommendation**		**Negative recommendation**	
1	Strong recommendation for the intervention	1-	Strong recommendation against the intervention
2	Limited recommendation for the intervention	2-	Limited recommendation against the intervention
3	Weak recommendation for the intervention	3-	Weak recommendation against the intervention
4	No recommendation possible		

The treatment recommendations are summarised in Table 3.

**Table 3.** Summary of recommendations for the treatment of PTSD (Bandelow et al. 2023b).

Treatment	Recommendation	LoE	RG
***Medications***			
SSRIs (fluoxetine, paroxetine, sertraline)	Fluoxetine, paroxetine, and sertraline are first-line treatments for PTSD	A	1
SNRI venlafaxine	Venlafaxine is a first-line treatment for PTSD	A	1
TCA amitriptyline	Amitriptyline is effective in PTSD. It is associated with more side effects than SSRIs/SNRIs	A	2
TCA imipramine	Imipramine was effective in DBPC studies; however, it was less effective than phenelzine in one study and as effective as phenelzine in an underpowered study	B	2
Irreversible MAOI phenelzine	Phenelzine was effective in DBPC studies; it was more effective than imipramine in one study and as effective as imipramine in an underpowered study	A	2
NaSSA mirtazapine	Mirtazapine was effective in one DBPC study	B	2
Antipsychotic risperidone	Risperidone is effective in PTSD according to DBPC studies. Due to increased adverse effects, it should only be used when standard treatments have failed or have not been tolerated	A	3
Antipsychotic quetiapine	Quetiapine was effective in one DBPC study. Due to a higher rate of adverse effects, it should only be used when standard treatments have failed or have not been tolerated	B	3
Anticonvulsant lamotrigine	Lamotrigine was effective in PTSD according to a placebo-controlled study. Due to its adverse effects, it should only be used when standard treatments have failed or have not been tolerated	B	3
Benzodiazepine analogue eszopiclone	Eszopiclone was effective in PTSD according to a DBPC study.	B	3
α_2A_ blocker prazosin	Prazosin was effective in treating nightmares in PTSD	A	2*
NMDA receptor antagonist ketamine	Ketamine infusion was superior to midazolam infusion	B	3
***Medications for treatment-unresponsive patients***			
Olanzapine	Olanzapine was effective in treatment-unresponsive patients with PTSD according to a DBPC study. Due to increased adverse effects, it should only be used when standard treatments have failed or have not been tolerated	B	3**
Olanzapine add-on to SSRIs	Olanzapine add-on to SSRIs was effective in treatment-unresponsive patients with PTSD according to a DBPC study. Due to increased adverse effects, it should only be used when standard treatments have failed or have not been tolerated	B	3**
Risperidone add-on to SSRIs	Risperidone add-on to SSRIs was effective in treatment-unresponsive patients with PTSD according to a DBPC study. Due to increased adverse effects, it should only be used when standard treatments have failed or have not been tolerated	B	3**
***Medications for secondary prevention of PTSD***			
Hydrocortisone	Hydrocortisone was effective in placebo-controlled studies	A	2
Benzodiazepines	The prophylactic administration of benzodiazepines was not effective in preventing PTSD	A-	1-
Escitalopram	The prophylactic administration of escitalopram was not effective in preventing PTSD	A-	1-
Propranolol	The prophylactic administration of propranolol was not effective in preventing PTSD	A-	1-
Gabapentin	The prophylactic administration of gabapentin was not effective in preventing PTSD	B-	2-
Oxytocin intranasal	The prophylactic administration of intranasal oxytocin was not effective in preventing PTSD	B-	2-
***Psychotherapy***			
Cognitive behavioural therapy (CBT)/Narrative Exposure Therapy (NET)	Evidence for the superiority of CBT and NET for PTSD to active controls is inconsistent, with many studies showing no difference. The meta-analysis of CBT/NET vs. active controls results showed a medium effect size. However, effect sizes may have been overestimated, as indication for publication bias was found.	A	1
Dialectical behavioural therapy (DBT)	DBT was more effective than CBT in one study	B	2
Internet interventions based on CBT (iCBT)	iCBT was superior to waitlist in some, but not all studies, and not superior to active controls (psychological placebo, TAU)	A-	1-
Virtual reality exposure (VRE)	VRE was more effective than waitlist and as effective as imaginal exposure	B	2
EMDR	EMDR was superior to waitlist in some, but not all studies. It was superior to psychological placebo and pill placebo, but not superior to psychological placebo or TAU. Comparisons with CBT are inconclusive	A-	1-
‘Debriefing’	Immediate psychotherapy after trauma (debriefing) is not effective and may worsen symptomatology	A-	1-
***Combination of psychotherapy and medication***			
CBT vs. drug	CBT was as effective as drug treatment in one study	B	2
Combination vs. CBT alone	There is inconsistent evidence whether the combination of CBT + medication is more effective than CBT alone	D	3
Combination vs. drug alone	There is inconsistent evidence whether the combination of CBT + medication is more effective than medication alone	D	3
***Enhancing exposure/virtual reality exposure***			
D-cycloserine + exposure	Using D-cycloserine for enhancing exposure therapy is not effective	B-	2-
***Neurostimulation***			
Repetitive transcranial magnetic stimulation (rTMS)	rTMS was effective in one sham-controlled study	B	2

* Only for treating nightmares in PTSD.

** Only for treatment-unresponsive patients.

LoE, level of evidence (see Table 2 for explanation).

RG, recommendation grade.

### Psychological treatment

Psychotherapy is a widely recognised treatment for individuals suffering from PTSD. Beyond formal psychotherapy, individuals who have experienced severe trauma require supportive conversations with healthcare providers, family members, and friends.

#### Cognitive-behavioural therapy (CBT)

“CBT” was used as an umbrella term for a range of different therapeutic approaches, including trauma-focused CBT, cognitive processing therapy, stress inoculation training, prolonged exposure, cognitive restructuring and imagery modification, and mindfulness-based cognitive therapy, among others. CBT is often used in the form of “trauma-focused” treatment. In a typical trauma-focused intervention the patient is confronted with the traumatic stimuli, requested to describe his traumatic experience and relieve it in imagination in order to achieve desensitisation of trauma-related cues. In addition, the patient is requested to practice *in vivo* exposure (e.g. driving to a place where a car accident happened).

Several included studies have evaluated Narrative Exposure Therapy (NET), a variant of trauma-focused CBT that involves the reconstruction of episodic traumatic memories through structured biographical narration with an emphasis on traumatic experiences. However, despite its empirical evaluation, this intervention—originally developed for use in third-world countries—is not widely implemented in routine clinical practice.

A substantial proportion (61%) of the evaluated CBT studies did not meet established scientific standards used by this guideline, because they relied exclusively on a waitlist control condition, compared psychotherapy with a reference treatment in a study lacking adequate statistical power, or employed a non-validated therapeutic method as the comparator, i.e., a method lacking confirmation from previous randomised controlled trials.

Many studies comparing CBT with an active control condition did not find significant differences between the groups. In 6 studies, CBT did not even outperform the waitlist control. Nonetheless, meta-analytic results indicate that CBT demonstrates a statistically significant advantage over active control conditions, with a medium effect size observed (Cohen’s *d* = 0.64). The meta-analysis revealed a substantial indication of publication bias, suggesting that the effect sizes reported in published RCTs may overestimate the true efficacy of CBT. Following adjustment for publication bias using Duval and Tweedie’s trim-and-fill method, the effect size was reduced to 0.35.

#### Digital CBT-based interventions (iCBT)

In the recent years, increasingly more studies have been conducted with CBT delivered *via* computers or smartphone in which the patients have little or no contact with therapists.

Some trials have shown higher efficacy of iCBT when compared with waiting list controls. However, two studies comparing iCBT with waitlist or another inactive control group were negative, and two comparisons with active control conditions also failed to show superiority. Thus, the current database does not support iCBT for PTSD.

#### Virtual reality exposure therapy

Exposure therapies using virtual or augmented reality settings have also gained increasing interest in the past years. Virtual reality exposure therapy was superior to waitlist and as effective as imaginal exposure treatment in one study. Another study showing it is as effective as *in vivo* exposure was underpowered.

#### Eye movement desensitization and reprocessing therapy (EMDR)

The core of EMDR involves having the patient recall traumatic events while following the movements of the therapist’s fingers. However, it is unclear whether these eye movements are a necessary ingredient of the treatment, as the only studies comparing EMDR with or without eye movements did not find a difference in efficacy (Boudewyns and Hyer 1996; Devilly et al. 1998). In both of these studies, neither EMDR nor EMDR without eye movements were superior to controls.

According to recommendations provided by proponents of EMDR, the eye movement technique may be substituted by other forms of bilateral stimulation, such as alternating auditory tones, tactile taps to both ears, or knee tapping. However, there is no empirical evidence to support the notion that the eye movement technique in EMDR can be freely substituted with other forms of stimulation.

Theories on the putative mechanism of action (for example, “bilateral stimulation enhances the communication between the left and right hemisphere of the brain”) remain pseudo-scientific narratives rather than evidence-based causal explanations. It is difficult to accept, from a scientific perspective, that simple activities such as producing noises in both ears or tapping on the knees of a patient could alter neural circuits and alleviate the debilitating symptoms associated with PTSD.

The reviewed trials do not provide sufficient evidence to support a recommendation of EMDR for PTSD. Most comparisons relied on waitlist control groups, and one of these studies reported null findings. The meta-analysis found no significant differences in efficacy between EMDR and active control conditions, suggesting that the observed effects of this intervention may be attributable to placebo mechanisms.

Comparisons with CBT yielded heterogeneous findings, with some studies indicating lower efficacy of EMDR and others suggesting superior outcomes. The majority of comparisons reporting comparable efficacy between the two interventions were insufficiently powered for non-inferiority analyses.

In contrast to the present findings, some meta-analyses and clinical guidelines recommend EMDR (American Psychological Association 2017; Bisson et al. 2019; Roberts et al. 2019; Lewis et al. 2020). However, these reviews did not distinguish between active and inactive control conditions.

#### Early psychological interventions

“Critical incident stress debriefing”—a therapeutic conversation conducted immediately with individuals following a traumatic event—has been implemented in efforts to prevent PTSD. This approach entails providing all trauma-exposed individuals with immediate psychotherapy aimed at prevention. However, only a relatively small proportion of trauma survivors ultimately develop PTSD. Universal intervention would incur considerable cost and time, and may, in some cases, provoke PTSD in individuals who otherwise would not have developed the disorder. Many healthy individuals experience a natural remission of symptoms over time, but repeated exposure to trauma-related content during such sessions may interfere with this spontaneous recovery process.

Our analysis of debriefing studies indicated that the intervention did not differ from inactive control conditions and, in some cases, was associated with poorer outcomes compared to no treatment.

Other meta-analyses of debriefing also showed that it did not improve natural recovery from trauma-related disorders (van Emmerik et al. 2002; Roberts et al. 2019), and other guidelines, e.g. ref (Phelps et al. 2022), also advise against the use of debriefing.

#### Psychodynamic therapy

Although psychoanalytic therapy (psychodynamic therapy) claims to effectively treat psychological trauma, no randomised clinical trial meeting the scientific standards of clinical practice guidelines has been found for the treatment of PTSD using this approach.

#### Long-term psychological treatment

Most trials with psychological treatments are of relatively short duration (e.g. 8–24 weeks). There is a lack of controlled studies comparing the efficacy of shorter versus longer treatment durations (e.g. 12 weeks vs. 24 weeks). As a result, evidence-based recommendations for the long-term treatments commonly used in routine practice (e.g. one year or more), cannot be made.

### Pharmacological treatments

Pharmacological treatment options for PTSD are summarised in Table 3. Recommended doses are listed in Table 4.

**Table 4.** Dose ranges for pharmacological treatment of PTSD (Bandelow et al. 2023b).

Treatment	Drug	Dose Range for Adults
*Selective serotonin reuptake inhibitors (SSRIs)*	Fluoxetine	20–40 mg
	Paroxetine	20–60 mg
	Sertraline	50–200 mg
*Serotonin noradrenaline inhibitor (SNRI)*	Venlafaxine	75–225 mg
*Tricyclic antidepressants (TCA)*	Amitriptyline	75–150 mg
	Imipramine	75–250 mg
*Benzodiazepines*	Alprazolam	1.5–8 mg
	Bromazepam	1.5–6 mg
	Clonazepam	1–4 mg
	Diazepam	5–20 mg
	Lorazepam	2–8 mg
*Noradrenergic and specific serotonergic antidepressant (NaSSA)*	Mirtazapine	30–60 mg
*Monoamineoxidase inhibitor (MAO)*	Phenelzine	45–90 mg
*Atypical antipsychotic*	Quetiapine	50–300 mg
*Anticonvulsant*	Lamotrigine	25–500 mg
*α_1_-Antagonist*	Prazosin	1–10 mg

The SSRIs *fluoxetine, paroxetine*, and *sertraline*, as well as the serotonin–norepinephrine reuptake inhibitor (SNRI) *venlafaxine*, may be considered first-line treatments.

The tricyclic antidepressants (TCAs) *amitriptyline* and *imipramine* are regarded as second-line options, as they are associated with a higher incidence of adverse effects, an increased risk of overdose, and lower adherence compared with SSRIs.

The monoamine oxidase inhibitor (MAOI) *phenelzine* is no longer used routinely and should be reserved as a second-line treatment because of its adverse effect profile and potential for severe drug interactions.

The antidepressant *mirtazapine* demonstrated efficacy in one placebo-controlled study.

The antipsychotic *risperidone* should be considered a third-line option due to the risk of adverse effects, including extrapyramidal symptoms and akathisia, even at doses lower than those used for schizophrenia. Similarly, *quetiapine* should be restricted to third-line use because of its association with metabolic adverse effects.

The anticonvulsant *lamotrigine* has been linked to rare but serious dermatological reactions, including Stevens–Johnson syndrome and toxic epidermal necrolysis, and should therefore be reserved for cases refractory to other treatments.

The cyclopyrrolone hypnotic (‘Z-substance’) *eszopiclone* was found to be effective in one DBPC study. Although Z-substances exhibit a lower risk of dependence than benzodiazepines, they are not recommended for long-term use.

In one DBPC study, infusion of the N-methyl-D-aspartate (NMDA) receptor antagonist *ketamine* was superior to midazolam infusion.

The α1-adrenergic antagonist *prazosin* has been shown in several studies—most reporting positive findings—to reduce nightmares in patients with PTSD; however, evidence for an effect on overall PTSD symptom severity was not established.

#### Long-term treatment

PTSD frequently follows a chronic course and therefore sometimes necessitates long-term medication treatment. Sustained efficacy has been demonstrated for the SSRIs fluoxetine and sertraline as well as for the SNRI venlafaxine. These trials, with durations ranging from 24 to 52 weeks, indicate that continuation of pharmacological treatment for at least one year is required to reduce the risk of relapse.

In the absence of RCTs evaluating lower maintenance dosages, it is recommended that the dosages used during the acute treatment phase be maintained during long-term therapy.

#### Prophylactic medication treatments

Prophylactic pharmacological interventions have been proposed to prevent the development of posttraumatic symptoms following exposure to major trauma. However, most medications evaluated for the immediate prevention of PTSD after trauma—including benzodiazepines, escitalopram, propranolol, gabapentin, and intranasal oxytocin—have not demonstrated efficacy. Hydrocortisone infusion showed beneficial effects in three studies. Nevertheless, a Cochrane review of pharmacological interventions for the secondary prevention of PTSD concluded that the available evidence does not support the use of any medication for preventing PTSD in individuals exposed to traumatic events, irrespective of the presence of psychological symptoms (Bertolini et al. 2022).

### Comparisons of psychotherapy and pharmacotherapy for PTSD and their combination

Only a limited number of studies have directly compared the efficacy of psychological and pharmacological interventions (Table 3). A single trial directly comparing CBT with paroxetine reported no significant differences between the two treatments. Evidence regarding the efficacy of combined treatment approaches remains heterogeneous. One study found that the combination of CBT and pharmacotherapy was more effective than CBT alone, whereas another observed no additional benefit from adding paroxetine to CBT. Conversely, one trial demonstrated superiority of combined treatment over pharmacotherapy alone, while another did not identify an incremental benefit of adding CBT to paroxetine. Although these findings are inconclusive, the overall pattern of evidence suggests a potential advantage of combined treatment over either modality administered in isolation.

Based on the assumption that the use of certain drugs that can facilitate extinction fear reactions could enhance the efficacy of exposure therapy especially for PTSD refractory to psychotherapy, D-cycloserine (DCS) and methylenedioxymethamphetamine (MDMA) were investigated in PTSD. DCS is an antibiotic that was introduced to treat tuberculosis and produces a facilitation of extinction of fear responses in rats. Four studies investigated the effects of augmenting exposure therapy or virtual reality exposure with D-cycloserine. Two studies reported no additional benefit, one reported a positive effect, and one observed a worsening of outcomes. Overall, the available evidence does not support a recommendation for this combined approach. The ‘recreational drug’ MDMA (‘Ecstasy’) was reported to decrease feelings of fear and produce euphoria, increased well-being, sociability and self-confidence while maintaining a clear-headed of state of consciousness. Therefore, it was investigated whether the drug could enhance the efficacy of psychotherapy. Five controlled studies reported efficacy of MDMA as an adjunct to psychotherapy in the treatment of PTSD. However, MDMA is associated with potentially dangerous adverse effects in cases of overdose, including hypertension, syncope, loss of consciousness, epileptic seizures, and hyperthermia. Moreover, as MDMA is not produced under the regulatory oversight of regulatory administrations, its clinical use cannot be recommended.

### Management of treatment-resistant PTSD

A meta-analysis reported a nonresponse rate of 39% for pharmacotherapy (Jia et al. 2025), while an equivalent nonresponse rate of 39% was observed for psychological treatments (Semmlinger et al. 2024). The nonresponse rate was even 45% in psychotherapy studies employing an intent-to-treat protocol. When pharmacotherapy is ineffective, clinicians can utilise numerous alternative medications or augmentation strategies to improve response rates. If a patient fails to show sufficient improvement through CBT, it is possible to switch to another approach within the spectrum of CBT-oriented techniques. However, there is a lack of studies on the use of alternative CBT techniques for cases of non-response to standard CBT.

Before treatment-resistance is assumed in a patient, it should be ensured that the medication treatment duration was adequate (at least 8 to 10 weeks) and that the dose was maximised within the recommended range.

Only three studies have exclusively examined patients with treatment-resistant PTSD. These studies support the use of olanzapine either as monotherapy or as an adjunct to selective serotonin reuptake inhibitors (SSRIs), as well as SSRI augmentation with risperidone.

Although switching studies are lacking, in cases where a medication is ineffective, other standard medications should first be tried, such as switching from an SSRI to an SNRI. Switching can also be tried within the same pharmacological class; for example, switching from one SSRI to another is appropriate, as these substances differ chemically.

Second-line pharmacological options may include amitriptyline, imipramine, mirtazapine, or phenelzine. Third-line treatments involve agents such as risperidone, quetiapine, lamotrigine, eszopiclone, or ketamine infusion. Treatment with antipsychotics may be associated with adverse effects, including metabolic syndrome (notably with olanzapine) and extrapyramidal symptoms or akathisia (commonly observed with risperidone), among other side effects.

CBT can be considered an alternative when pharmacotherapy is ineffective. Conversely, pharmacotherapy may be an option for patients who do not respond to CBT.

When a patient does not respond to any kind of treatment, malingering should be considered as a possible contributing factor. Nevertheless, treatment resistance also frequently occurs in individuals with authentic PTSD, especially among those who have experienced irreversible loss.

### Neurostimulation methods

Several studies investigated the efficacy of neurostimulation methods in PTSD.

#### Repetitive transcranial magnetic stimulation (rTMS)

rTMS is a non-invasive brain stimulation therapy that uses magnetic fields to stimulate nerve cells in specific areas of the brain. A coil placed on the scalp delivers repeated magnetic pulses that can influence brain activity. The magnetic pulses pass through the skull and induce small electrical currents in the brain which can either increase or decrease activity in these brain regions, e. g., the right dorsolateral prefrontal cortex (DLPFC). rTMS was effective in one controlled study (Cohen et al. 2004).

### PTSD in children and adolescents

Treatment recommendations for children and adolescents are summarised in Table 5.

**Table 5.** Recommendations for the treatment of PTSD in children and adolescents (Bandelow et al. 2023b).

Treatment	Recommendation	LoE	RG
***Medications***			
SSRI sertraline	Evidence for sertraline is inconclusive, with one study showing superiority over placebo and another one showing no difference to placebo	D	4
***Psychotherapy***			
CBT	Evidence for CBT for PTSD in children and adolescents is mixed. Some studies show superiority to waitlist, but several studies do not show a difference to waitlist or no intervention. Four studies show superiority to active controls. The meta-analysis of studies with active controls showed a significant difference between CBT and active controls (medium effect size)	A	1
EMDR	Evidence for EMDR is inconclusive. Two studies showed a difference to waitlist; two studies did not. Comparisons with active controls are lacking. Two studies showing equal efficacy of EMDR and CBT were underpowered	D	4
***Enhancing exposure with D-cycloserine***			
D-cycloserine + exposure	Enhancing exposure with adjunct D-cycloserine was not effective	B-	2-

LoE, level of evidence (see Table 2 for explanation).

RG, recommendation grade.

#### Psychotherapies for PTSD in children and adolescents

Although many studies of CBT demonstrated superiority over waitlist conditions, six comparisons with waitlist or no-intervention controls did not identify significant differences. In contrast, four comparisons with active control conditions reported favourable outcomes for CBT. A meta-analysis restricted to studies employing active controls demonstrated a significant advantage of CBT over comparator interventions, with a medium effect size (Cohen’s *d* = 0.73).

Evidence regarding the use of eye movement desensitisation and reprocessing (EMDR) for the treatment of PTSD in children and adolescents remains inconsistent. In two of four studies, EMDR was not superior to waitlist controls, and direct comparisons with active control conditions are currently lacking. Two studies reporting comparable efficacy of EMDR and CBT were underpowered.

One study comparing D-cycloserine- and placebo-augmentation of exposure therapy found no difference between in children with PTSD.

A newer meta-analysis of studies comparing the effect of Internet- and mobile-based interventions for posttraumatic stress symptoms in youths to control conditions found no difference (Schulte et al. 2024).

#### Pharmacological treatments for PTSD in children and adolescents

Only two placebo-controlled studies have evaluated sertraline in children and adolescents with PTSD (Table 5). One study did not demonstrate superiority over placebo, whereas the other reported that the addition of sertraline to CBT was more effective than placebo in combination with CBT.

## Discussion

PTSD is a chronic and disabling condition that can be effectively treated. Symptom improvement is often observed over time as distance from the traumatic event increases. However, a significant proportion of patients continue to experience symptoms despite receiving adequate psychotherapy and medication trials. A longer treatment duration and higher drug doses may be necessary in these cases, and several strategies exist for treatment-unresponsive cases.

Several myths surround PTSD, including the beliefs that nearly everyone who experiences a severe trauma inevitably develops PTSD, that all trauma-related disorders require treatment, that all victims of traumatic events must receive immediate prophylactic psychotherapy even if PTSD symptoms have not yet emerged, that psychotherapy is generally more effective than pharmacological treatments, and that eye movement desensitisation and reprocessing (EMDR) and psychodynamic therapy are treatments fully supported by empirical evidence. However, the review of the evidence presents a different picture.

In contrast to other guidelines on PTSD, the WFSBP guideline evaluated the efficacy of psychotherapies exclusively based on comparisons with active control groups (e.g. psychological placebo) rather than comparisons with waitlist controls. Whereas minimal improvement occurs in waitlist control groups, patients receiving psychological placebo treatments demonstrate significant symptom reduction (Bandelow et al. 2015). Medications would not receive approval without evidence of efficacy from double-blind placebo-controlled trials. If medication studies only required superiority to waitlist, numerous drugs with questionable efficacy would gain approval. Consequently, it is consistent to test psychotherapeutic interventions against their placebo equivalent, namely active control conditions.

CBT was found to be the psychotherapy modality with the best body of evidence, although the effect size in comparisons to active controls was small after correction for publication bias. Virtual reality exposure therapy may be an emerging treatment option for PTSD. Digital psychotherapeutic interventions based on CBT were not superior to controls.

Early prophylactic psychotherapy of trauma victims is not effective and may even be harmful.

EMDR, a therapeutic approach with an unexplored mechanism of action, is frequently promoted for the treatment of PTSD. However, current evidence does not indicate that the therapeutic effects of EMDR exceed those attributable to placebo or other non-specific factors.

Although psychodynamic therapy claims to effectively treat psychological trauma, no evidence from RCTs for the efficacy of this treatment approach for PTSD could be found. Nonetheless, psychodynamic therapy is widely used. For example, according to the German National Association of Statutory Health Insurance Physicians (KBV 2024), 33% of all psychological therapies are psychodynamic treatments, and 66% are CBT.

Among medications, SSRIs and the SNRI venlafaxine are first-line treatments. For treatment-unresponsive patients, several alternative medications exist.

Attempts have been made to provide prophylactic pharmacological treatment to individuals exposed to severe trauma to prevent the development of PTSD. However, there is currently no sufficient evidence to support the clinical utility of such prophylactic interventions.

Repetitive transcranial magnetic stimulation (rTMS) was effective in one study.

For children and adolescents with PTSD, CBT showed a medium effect size when compared to active controls. Studies of SSRI treatment showed inconsistent results. More controlled studies with children and adolescents are needed.

Virtual and augmented reality–based exposure therapies may represent potential future treatment options for PTSD.

Current evidence regarding digital CBT interventions delivered *via* computers or smartphones remains insufficient. Although existing digital psychotherapy programs demonstrate increasing sophistication, it is anticipated that the integration of artificial intelligence (AI) into such programs will significantly enhance these platforms’ communication capabilities and ability to provide personalised treatment.

In summary, PTSD can be effectively treated with CBT and pharmacotherapy, leading to significant improvements in patients’ quality of life despite the often disabling nature of the disorder.
